# Burkitt's lymphoma presenting as acute dacryocystitis in a 14-year-Old male with ataxia telangiectasia: A case report and review of the literature

**DOI:** 10.1016/j.ajoc.2025.102288

**Published:** 2025-02-24

**Authors:** Samin Khannejad, Sajad Mansourian, Amirhossein Aghajani, Zohreh Nozarian, Seyed Mohsen Rafizadeh

**Affiliations:** a School of Medicine, Shahid Beheshti University of Medical Sciences, Tehran, Iran; b Department of Oculo-facial Plastic and Reconstructive Surgery, Farabi Eye Hospital, Tehran University of Medical Sciences, Tehran, Iran; c Department of Pathology, Farabi Hospital, Tehran University of Medical Sciences, Tehran, Iran

**Keywords:** Burkitt lymphoma, Ocular adnexal lymphoma, Dacryocystitis

## Abstract

**Purpose:**

This report describes an atypical presentation of a case of Burkitt lymphoma in a pediatric patient with ataxia telangiectasia.

**Main observations:**

A 14-year-old boy with a history of AT was referred to our hospital with lower eyelid swelling and medial canthus abscess. On physical examination, movement of the left eye was limited in downgaze and adduction. Two mm proptosis was observed on the left side. Snellen's Visual acuity in the left eye was 8/20. The rest of the examination was normal. Magnetic resonance imaging (MRI) was performed and showed an infiltrative mass in the inferior of the left orbit, left ethmoidal sinus, and maxillary sinus. The patient subsequently underwent an incisional biopsy. The biopsy specimen was sent for histopathologic evaluation. Histopathology was significant for atypical monotonous cell infiltrations in the fibroconnective tissue and the presence of pleomorphic, irregularly shaped nuclei with multiple mitoses. Immunohistochemistry (IHC) findings were consistent with Burkitt's lymphoma, and the patient was referred to the oncology department for chemotherapy and appropriate treatment.

**Conclusion:**

and Significance

Orbital and lacrimal duct involvement is a rare presentation of Burkitt lymphoma especially in the pediatric population. In the new-onset nasolacrimal duct obstruction (NLDO) in a child or teenager, the differential diagnosis should include malignancies, especially leukemia/lymphoma infiltrations.

## 1 Introduction

Burkitt's lymphoma (BL) was first described by Denis Burkitt in 1958 in African children with presentations in the mandible. It is a type of non-Hodgkin, B-cell lymphoma with rapid progression.^1^^,^^2^ Lymphomas are the third leading cause of death in children. Non-Hodgkin's lymphomas (NHL) are the most frequent type.^2^ Extranodal lymphomas account for only five percent of head and neck neoplasms. However, the head and neck are the second most common area of involvement in such malignancies.^3^ According to previous reports, only 1–2% of NHLs occur in the orbital adnexal margin.^4^

The manifestations and epidemiology of Burkitt's lymphoma vary across different populations. In general, abdomen is the main site of involvement in NHL, followed by the head and neck. Furthermore, an association between NHL symptoms and age has been established. ^5^A study conducted by Levine et al., involving 421 cases, revealed a higher incidence of involvement in the nasopharynx, cervical lymph nodes, and ileum among younger patients.^5^ Additionally, constitutional symptoms manifest with greater frequency in adult patients.^6^ Primary nasolacrimal NHL is rare and may resemble dacryocystitis symptoms and gross morphology.^7^ Tumors of the lacrimal duct most commonly present with epiphora, refractory dacryocystitis, or lacrimal sac mass.^8^ So far, only seven cases of primary orbital and periorbital involvement with Burkitt's lymphoma have been reported in the pediatric population.

Here, we describe a case of Burkitt's lymphoma with symptoms similar to acute dacryocystitis to demonstrate the various clinical presentations of Burkitt's lymphoma and to suggest that physicians in practice should maintain a high level of awareness for malignancy when approaching similar conditions.

A 14-year-old male patient was referred to our hospital with symptoms of redness and swelling of the lower eyelid and medial canthus for one month, which did not respond to previous treatments. The patient was treated with ciprofloxacin drops and erythromycin ointment, intravenous ampicillin and vancomycin, and oral linezolid and co-amoxiclav before being referred by an ophthalmologist at another institution with a diagnosis of pre-septal cellulitis.

**Fig. 1:**
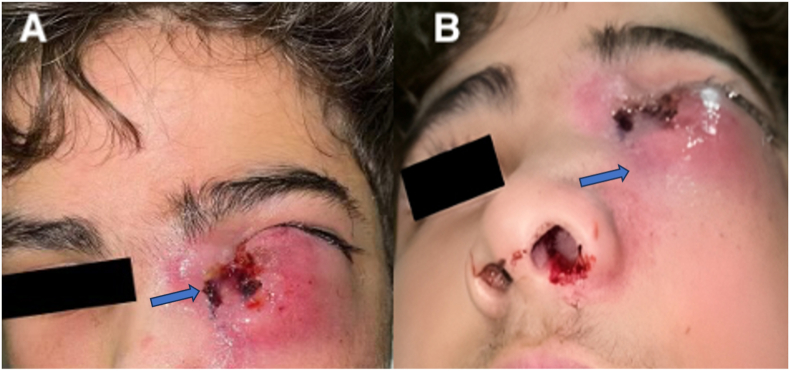
A, B: The patient's appearance: Swelling and ecchymosis of the lower lid of left eye.

Despite all the medications and hospitalization, the patient's symptoms continued with slight improvement and finally, he was referred to our center (Fig. 1). The patient did not mention any history of systemic symptoms such as fatigue, abdominal cramps, cough, or B symptoms such as night sweats, fever, or weight loss. His medical history was significant for ataxia telangiectasia (AT), which affected his mobility, and he was wheelchair dependent. The patient had no history of lacrimal symptoms before that. His ocular problems history was also negative. No other significant abnormalities were found on systemic examination.

A comprehensive ophthalmic examination was performed, and visual acuity was 20/20 in the right eye (OD) and 8/20 in the left eye (OS). The intraocular pressure was 14 (OD), and the examination of the left eye's intraocular pressure was impossible. Swelling and erythema were present in the inferior eyelid and left medial canthus. An ulcerative lesion was also present in the left medial canthus. Minus-2 limitation in downgaze and adduction was observed in ocular motility. The results of the funduscopic and slit lamp examination of both eyes were not significant. No local lymphadenopathy was observed. The results of laboratory tests, including complete blood count (CBC), were normal.

**Fig. 2:**
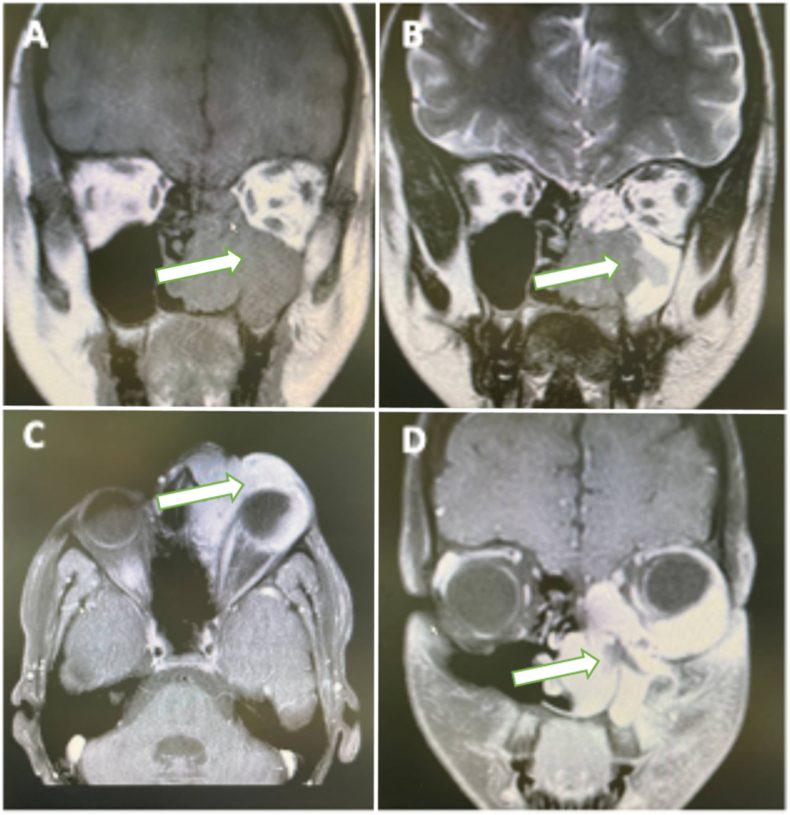
Coronal T1-weighted (A) and T2-weighted (B) MRI of paranasal sinuses showing an infiltrative mass in the left ethmoidal and maxillary sinuses extending to the right ethmoidal sinus and inferior surface of the left orbit. T1-weighted orbital MRI with gadolinium enhancement, axial (C) and coronal (D) view display a significant enhancement.

The patient's symptoms were consistent with acute dacryocystitis with orbital involvement. Further assessment, including imaging studies, was conducted. The patient's neurologist prohibited computed tomography (CT) scans due to a positive history of AT. Accordingly, magnetic resonance imaging (MRI) was performed, which revealed an infiltrative mass in the inferior surface of the left orbit, ethmoidal sinus, and maxillary sinus (Fig. 2).

**Fig. 3:**
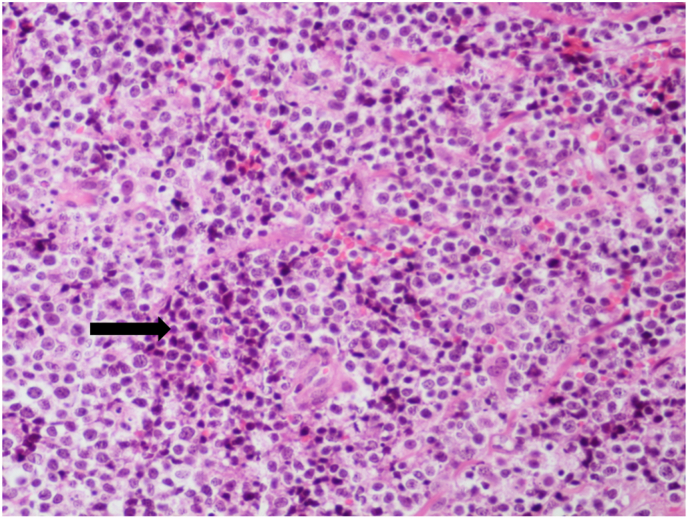
H&E-stained slide showed diffuse and monotonous proliferation of atypical mononucleated cells arranged in sheets (X400).

**Fig. 4:**
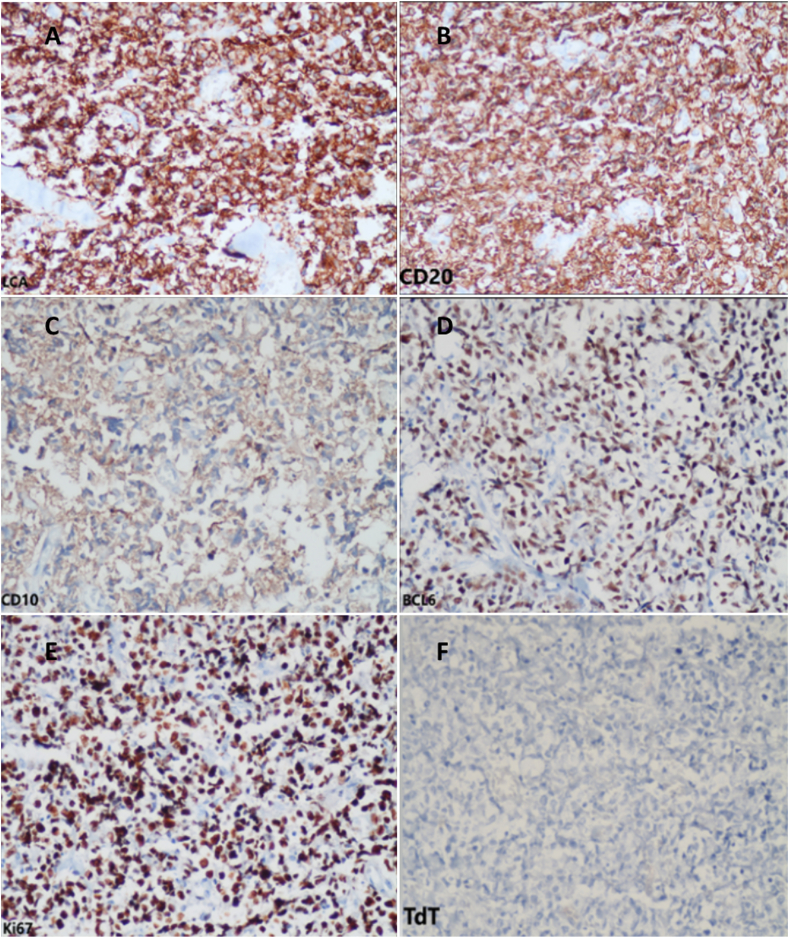
IHC findings, including positive LCA (A), CD20 (B), and BCL6 (D), and CD10 with high proliferative activity Ki-67 near to 100 % (E) and negative TdT (F).

Our patient went under general anesthesia, and an incisional biopsy was obtained to diagnose the pathology of the orbital mass. Necrosis was observed during surgery. A biopsy was taken from the necrotic area and its surroundings. The specimen was properly fixed in formalin and sent to the pathology laboratory for evaluation. Histopathology revealed fibrous connective tissue diffusely infiltrated by monotonous medium-sized atypical cells with indistinct cytoplasmic borders, pleomorphic nuclei, some with angular appearance, multiple small nucleoli, and numerous mitotic figures. No starry sky appearance was evident in histopathologic slides (Fig. 3). Histopathologic findings raised suspicion for high-grade lymphoma. Furthermore, an IHC panel was conducted to assess differential diagnoses associated with round cell malignancies. The panel included LCA, CD20, CD3, CD30, BCL6, BCL2, CD10, and CD99 to exclude the possibility of PNET/Ewing sarcoma. TDT staining was performed to eliminate the likelihood of Lymphoblastic Leukemia, MPO staining was carried out to rule out Myeloid Leukemia, and desmin and myogenin staining were done to rule out Rhabdomyosarcoma. Additionally, chromogranin, synaptophysin (to rule out neuroblastoma), and Ki-67 were included in the panel for further diagnostic assessment. IHC was performed using the Mouse/Rabbit PolyVue PlusTMHRP/DAB Detection System with primary antibodies (Diagnostic BioSystems Inc). LCA, CD20, BCL6, and CD10 were positive with high proliferative activity (Ki-67 status 100 %). Other markers were negative. Histopathologic findings and IHC results were consistent with high-grade B-cell lymphoma, mostly suggesting Burkitt's lymphoma (Fig. 4).

The parents were fully informed about the nature of the disease and its prognosis. Later, the patient was admitted to the oncology ward in another hospital for additional observations and chemotherapy. At the time of writing, the patient had merely received only one course of chemotherapy with COMP regimen, including cyclophosphamide, vincristine, methotrexate, and prednisone. At the follow-up appointment, after two months, his eye swelling and other symptoms improved significantly on macroscopic examination.

## 2 Discussion

The present report describes a 14-year-old boy, a known case of AT, who presented with left periorbital swelling and dacryocystitis due to Burkitt lymphoma with orbital and lacrimal drainage system involvement. Burkitt lymphoma (BL) stands as a rare and highly aggressive subtype of non-Hodgkin lymphoma, impacting around 1500 individuals annually.

The endemic form of BL was initially identified in regions with a high prevalence of malaria, including sub-Saharan Africa, and was found to be associated with the Epstein-Barr virus in almost all of the cases. BL is the most common childhood malignancy in sub-Saharan Africans unlike in other regions. The sporadic form is seen in Western Europe and the United States and is also found to be associated with Epstein-Barr Virus; however, Epstein-Barr Virus exists much less frequently in BL cases. In the sporadic form the main site of involvement is the abdomen. However, the most common site for endemic BL was found to be the face, including the jaw. Moreover, bone marrow and lymph node involvement are more common in the United States,.^9^ In Caucasians, BL primarily occurs in the sporadic form, and in Asia, despite the similar EBV prevalence to that of other regions, BL incidence is surprisingly lower. Notably, it has been found that the EBV in Asia is genetically different from that in Africa. However, EBV shares genomic similarities between America and Africa.^10^, ^11^, ^12^, ^13^, ^14^

The mean age of diagnosis of Burkitt lymphoma is 35.5 years, with three peaks at 10, 40, and 70 years according to previous reports.^15^ Moreover, sporadic BL mainly affects the abdomen (60–80 %), followed by the head and neck. Bone marrow involvement occurs in nearly 20 % of patients, and CNS involvement is rare. Tonsils, paranasal sinuses, nasopharynx, and oropharynx are the main affected sites in the head and neck. In addition, cervical adenopathy is a common finding.^16^, ^17^, ^18^ Non-Hodgkin's lymphoma rarely occurs in the orbital area, accounting for less than 5 % of cases.^19^ However, B-cell lymphomas account for the majority of orbital lymphomas (97 %), such as extranodal marginal zone B-cell lymphoma (EMZL) (53 %), diffuse large B-cell lymphoma (23 %), follicular lymphoma (9 %), and mantle cell lymphoma (5 %).^20^ Additionally, to the best of our knowledge only one case of pediatric lymphoma with lacrimal sac involvement, who was diagnosed with mucosal-associated lymphoid tissue (MALT) lymphoma, has been reported in the literature.^20^

The present case suffered from AT, an autosomal recessive disorder with mutations in the ATM protein-coding gene. AT manifests with systemic symptoms, including progressive ataxia, oculomotor apraxia, or extrapyramidal symptoms. It is well known that the occurrence of Burkitt's lymphoma is significantly associated with immunodeficiency status, as the incidence of this tumor is markedly higher in the immunocompromised population.^21^ Consistent with this, AT induces an immunodeficiency state as a significant predisposing factor for Burkitt lymphoma. Furthermore, individuals with AT are prone to developing lymphoid malignancies, possibly attributed to the crucial role of the ATM protein in DNA repair.^22^ Malignancy stands as a prominent factor contributing to mortality, second only to pulmonary diseases. The presence of a singular solid tumor in individuals with AT should prompt consideration and suspicion of potential underlying malignancies.^23^

In this regard, almost 40 % of patients with AT develop malignancies with a lymphoid predominance (80 %). This correlation may be due to genetic translocations and inversion mutations.^2^^,^^24^ In terms of clinical presentation, due to the progressive nature of Burkitt lymphoma, chronic symptoms, such as weight loss, generalized lymphadenopathy, or cachexia are less common. However, ascites, jaundice, hepatosplenomegaly, and fever are more common in these patients.^25^ However, in orbital involvement, similar to the present case, the most common symptoms are orbital swelling, decreased motility, pain, proptosis, or chemosis. Accordingly, these symptoms are often nonspecific. Therefore, diagnostic incisional biopsy and early imaging studies could significantly aid in early diagnosis. However, the diagnosis of lymphoma must be confirmed by surgical biopsy.^1^^,^^20^^,^^26^ The t(8;14)/MYC-IGH fusion is the primary genetic defect in Burkitt lymphoma, usually confirms the diagnosis of Burkitt lymphoma; however, c-MYC translocation is not specific, and t(2;8) and t(8;22) are less frequently present.^27^ However, MYC IHC marker and molecular testing were not available in our center.

B-cell markers, including CD22, CD19, CD79a, CD20, Pax5, and germinal center markers, such as CD10 and BCL-6, are expressed in Burkitt lymphoma. The classic “starry sky” pattern, or dispersed layers of monomorphic B cells with medium size, could also be seen in Burkitt lymphoma.^28^ In the reported case, the histopathologic results and IHC findings were suggestive of BL.

In terms of treatment, chemotherapy is the mainstay for Burkitt's lymphoma and is highly effective. Over the years, many efforts have been made to optimize the treatment protocol and minimize the associated toxicities, with remarkable results.^29^^,^^30^ Event-free survival was 81.8 % in patients who received short-course chemotherapy regimens.^31^ In contrast to the older population, treatment outcomes in children have improved significantly due to improvements in the treatment regimen.^21^ Central nervous system or bone marrow involvement, residual disease despite treatment, and poor response to chemotherapeutic agents are related to poor prognosis in Burkitt lymphoma. Adding rituximab to the chemotherapy treatment plan has been associated with improved outcomes.^32^ Patients with AT also experience less favorable outcomes than the normal population due to treatment-related toxicity. The present case was referred to the oncology department and received chemotherapy accordingly.

Primary orbital and periorbital Burkitt's lymphoma is rare in the pediatric population. As far as we know, only seven cases of such disease have been previously reported (Table 1).^33^, ^34^, ^35^, ^36^, ^37^ In these cases, swelling, proptosis, globe displacement, chemosis, and decreased motility were the most common symptoms. A surgical biopsy was performed in all of these patients, and chemotherapy together with surgery or alone was the choice of treatment. Only two of these seven patients received radiation therapy. Two of them died, and the rest responded well to chemotherapy and had remissions. On the other hand, drainage system involvement is extremely rare in children, and previous literature mainly reported in the elderly population.^15^

**Table 1:** Literature review of prior cases.

Author, year	Sex,Age	Presentation and examination findings	Systemic involvement	Chemotherapy/radiotherapy/surgical intervention	Outcome	Symptom duration (days)	Stage
Bouali et al. (2016)	F, 2 years	Painless swelling, right eye proptosis	None	Cyclophosphamide, vincristine, methotrexate, and prednisone	The patient died after 6 months	28	1
Gupta et al. (2012)	M, 13 years	Proptosis, chemosis, eyeball displacement, cheek swelling, neck mass	Secondary lymphomatou-s deposits in the spine, retroperitoneu-m, liver, kidneys	Cyclophosphamide, vincristine, prednisone, intrathecal methotrexate, and radiotherapy	NA	20	NA
	M, 10 years	Proptosis, chemosis, subconjuncti-val haemorrhage, Nasal and ear discharge, B symptoms	NA	NA	The patient was in remission at 3,6 and 12 months follow up	10	NA
	M, 8 years	Right eye proptosis	Kidneys, pancreas, and lungs	Cyclophosphamide, Vi-ncristine, prednisone, doxorubicin, Methotr-exate	The patient passed after 17 months	NA	Stage 4
Grasso et al. (2016)	M, 3 years	Sever proptosis and chemosis with eyeball displacement	NA	Prednisone, vincristine, cyclophosphamide and rituximab, surgical intervention	The patient was well at one month follow up	7	Stage 4
Edelstein et al. (1997)	M, 26 months	Oral thrush, unilateral painless eye proptosis and swelling	Maxillary sinus, retroperitoneal lymphadenopathy, kidney, pancreas, liver	Cyclophosphamide, vincristine, prednisolone and doxorubicin	Patients' symptoms improved at 8 months	NA	NA
Arepalli et al. (2019)	M, 7 months	Periorbital, and cheek swelling, ecchymosis	No	Multimodal and triple intrathecal chemotherapy, Immunotherapy with anti-CD20 monoclonal antibody and rituximab	Symptoms improved at follow-up	NA	NA

## 3 Conclusion

Although extremely rare, Burkitt's lymphoma with orbital or lacrimal involvement may present as dacryocystitis with non-specific manifestations such as periorbital swelling. In general, in cases of new-onset dacryocystitis in children and especially in patients who already have immunodeficiency conditions, orbital and lacrimal system involvement with leukemia-lymphoma infiltration should be considered as a differential diagnosis according to the disparate approaches regarding the diagnosis and treatment period.

## CRediT authorship contribution statement

**Samin Khannejad:** Writing – original draft, Supervision, Project administration, Investigation. **Sajad Mansourian:** Writing – original draft, Project administration, Investigation. **Amirhossein Aghajani:** Writing – original draft, Project administration, Investigation. **Zohreh Nozarian:** Writing – review & editing, Writing – original draft, Investigation. **Seyed Mohsen Rafizadeh:** Writing – review & editing, Writing – original draft, Supervision, Project administration, Investigation, Data curation.

## Patient consent

Written informed consent was obtained from the parents for participation in the study and publication of this report and linked photos.

## Authorship

We attest that all of the authors had notable contribution to creation of the current manuscript.

All of the authors meet the ICMJE criteria.

## Declaration of generative AI and AI-assisted technologies in the writing process

None.

## Funding

This research received no specific grant from any funding agency in the public, commercial, or not-for-profit sectors.

## Declaration of competing interest

The authors declare that they have no known competing financial interests or personal relationships that could have appeared to influence the work reported in this paper.
